# Correlations between Microstructure Characteristics and Mechanical Properties in 5183 Aluminium Alloy Fabricated by Wire-Arc Additive Manufacturing with Different Arc Modes

**DOI:** 10.3390/ma11112075

**Published:** 2018-10-24

**Authors:** Xuewei Fang, Lijuan Zhang, Guopeng Chen, Xiaofeng Dang, Ke Huang, Lei Wang, Bingheng Lu

**Affiliations:** 1 The State Key Laboratory for Manufacturing Systems Engineering, Xi’an Jiaotong University, No. 99 Yan Cheung Road, Xi’an 710054, China; fangxuewei0801@163.com (X.F.); ke.huang@xjtu.edu.cn (K.H.); 2 National Innovation Institute of Additive Manufacturing, Buiding A, Door of Metropolis, Jinye Road, Gaoxin District, Xi’an 710065, China; ljz201709@126.com (L.Z.); chenguopeng@niiam.cn (G.C.); dangxiaofeng@niiam.cn (X.D.); wlei292@xjtu.edu.cn (L.W.); 3 Collaborative Innovation Center of High-End Manufacturing Equipment, Xi’an Jiaotong University, No. 99 Yan Cheung Road, Xi’an 710054, China

**Keywords:** additive manufacturing, cold metal transfer, mechanical property, porosity, microstructure, 5183 aluminium alloy

## Abstract

The effect of arc modes on the microstructure and tensile properties of 5183 aluminium alloy fabricated by cold metal transfer (CMT) processes has been thoroughly investigated. Heat inputs of CMT processes with three arc modes, i.e., CMT, CMT advance (CMT+A), and CMT pulse (CMT+P), were quantified, and their influence on the formation of pores were investigated. The highest tensile strength was found from samples built by the CMT+A process. This agrees well with their smallest average pore sizes. Average tensile strengths of CMT+A arc mode-built samples were 296.9 MPa and 291.8 MPa along the horizontal and vertical directions, respectively. The difference of tensile strength along the horizontal and vertical directions of the CMT+P and CMT samples was mainly caused by the pores at the interfaces between each deposited layer. The successfully built large 5183 aluminium parts by the CMT+A arc mode further proves that this arc mode is a suitable mode for manufacturing of 5183 aluminium alloy.

## 1. Introduction

In the past two decades, additive manufacturing (AM) has gained more and more attention in the manufacturing industry, especially in creating models and prototypes. This technology has now begun to emerge as an important commercial manufacturing method [1]. AM can promote an increase to design freedom, and decrease part cost by reducing material wastage and lead time. Therefore, weight-saving and complex parts can be economically produced [2]. Research interests in AM have focused on fabricating complex-shaped metal components that cannot be produced using traditional approaches. By far, the majority of AM researches have been focusing on the powder-bed-based selective laser melting and electron beam melting processes with different materials, such as Titanium alloy, Ti-based composites, Ni-based superalloy, Aluminium alloys and Al-based composites [3,4]. These alloys have high mechanical properties and have been used in various fields. However, the low material utilization and deposition rates always limit the application of these metals in building large parts. An alternative process, wire-arc additive manufacturing (WAAM), was reported by Williams et al. [5] as a suitable method for fabricating large metallic components with low cost and short lead time. WAAM has been recognized as one of the most efficient processing methods, which allows the fabrication of large components with sizes up to several meters, such as cruciform structures, stiffened panels, and wing ribs [6].

Aluminium alloys are widely used in various fields such as aerospace, ship building, and automobile industries, owing to their high-strength, light weight, and excellent corrosion resistance [7,8]. The traditional machining process of aluminium alloys parts needs specific equipment and generally causes huge wastage of raw materials. The Buy-to-Fly ratio may be as high as 10 to 1 in aviation manufacturing of aluminium alloy parts. Therefore, researchers have been seeking for new manufacturing methods to save resources in these years. WAAM has been considered as a promising method to improve material utilization and for cost saving. Therefore, a great deal of attention has been paid to the WAAM technology, which has a high deposition rate and requires low equipment investment [9]. Typical wire-based deposition methods such as gas metal arc welding (GMAW), gas tungsten arc welding (GTAW), and plasma arc welding (PAW) are popular due to their low costs and they have been applied to fabricate metallic parts [10,11,12].

To improve the heat input and energy density of the GMAW process, a modified metal inert-gas (MIG) welding process named cold metal transfer (CMT) was invented by Fronius Company [13]. The CMT process, which is a relatively new method, is characterized by its low heat input and high deposition rate. Moreover, CMT is a spatter-free dip transfer process via precisely controlling of current parameters and wire movements during the short-circuit transfer. The wire tip in the specially designed torch controlled by a powerful motor can reach a high frequency of forward and backward movement (up to 130 Hz) during welding. This can reduce the energy input to a minimum degree [14].

The CMT process is suitable to weld aluminium alloys due to its low heat input controlled by the back retreatment of wire filler technology [15]. The 5356 aluminium parts made by variable polarity GTAW were reported by Wang and Kovacevic [16] previously. The correlation between the geometric sizes of the deposited layer and building parameters was analysed, but the mechanical properties of the fabricated parts were not given. To predict the inter-pass temperature evolution, Geng et al. [17,18] developed a mathematical model for the GTAW process, which can be used to optimize the geometric appearance. The ultimate tensile strength and elongation of as-deposited 5A06 alloy samples made by GTAW are 273 MPa and 34%, respectively. Fang et al. [19] reported the effects of different arc modes on porosity formation and mechanical properties in printing of 2319 aluminum alloy by CMT. The variable polarity CMT process was employed to fabricate Al-6Mg alloy thin-wall by Zhang et al. [20]. The mechanical properties were then analyzed but the difference between various arc modes was not studied. It was proved by Gu et al. [21] that cold rolling is beneficial for decreasing micro-pores in the 2319 and 5087 aluminum alloys fabricated by pulse advanced CMT, and improve the tensile strength. However, the above-mentioned work addressed neither the effect of CMT arc modes on 5xxx aluminum alloy, nor their mechanical and microstructure characteristics.

In this paper, the 5183 aluminium wire was chosen as the raw material to systematically investigate the effect of different CMT arc modes on the porosity, microstructure, and mechanical properties of the WAAM materials. The main objective of this study was to provide a fundamental understanding of microstructure evolution involved in the CMT-based AM method with interest on the relationships among heat input, porosity, microstructures, and mechanical properties of 5183-Al fabricated by WAAM process. Finally, several aluminium parts were fabricated by the tuned processing parameters. This will be very significant for selecting the most suitable method to fabricate aluminum alloys by WAAM technology, which can improve material utilization and save costs compared with traditional methods, especially for large aluminium structural parts in aerospace industry.

## 2. Experimental Methods

**Figure 1:**
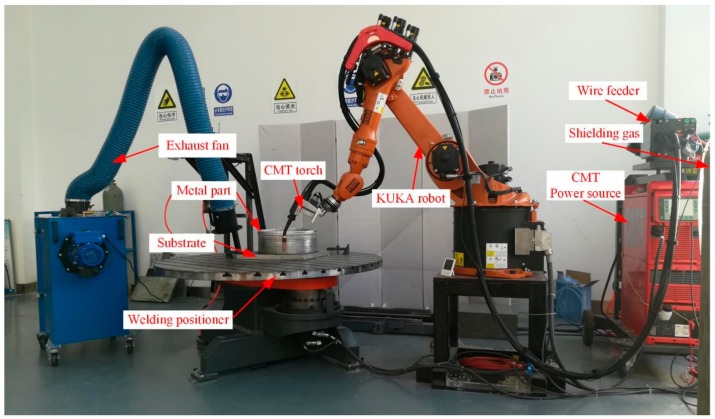
Set-up of cold metal transfer (CMT)-based wire-arc additive manufacturing (WAAM) system.

The set-up of the WAAM system in this research as shown in Figure 1 consists of a Fronius CMT+Advanced (CMT-A) system coupled with a VR-1550 wire feeder (Fronius, Pettenbach, Austria) and a KUKA KR16 robot (KUKA AG, Augsburg, Germany). The torch was mounted on the robot arm moving in the designated path to fabricate metal parts. The data of arc voltage and current were acquired by NI acquisition card, which was controlled by the SIMENS PLC units. The filler wire used in this research was 5183-Al with a diameter of 1.2 mm from Alcotec Wire Corporation (Traverse City, MI, USA). The base metal was 12 mm-thick 5083-H112 plate. The composition of the substrate and the filler wire are shown in Table 1. Before deposition, the surface of the substrate was cleaned by angle grinder, and then washed by acetone and alkaline solution, together with a subsequent drying process. 

**Figure 2:**
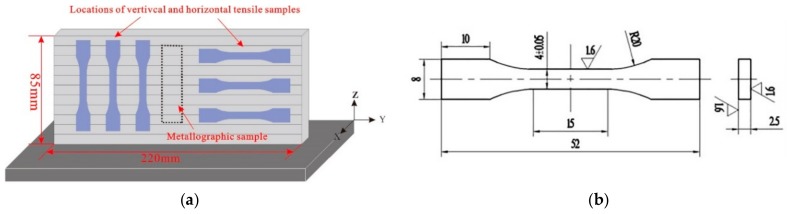
(**a**) Schematic graph of the sampling positions; (**b**) the dimensions of tensile sample.

The parameters, including wire feeding speed (5 m/min), travel speed (0.5 m/min), and shielding gas flow (25 L/min, 99.99% Ar), were kept as constant during deposition for the three arc modes. After deposition, the as-deposited blocks were characterized to examine the morphology and the compositions of the second phase particles. Specimens for tensile testing and metallographic observation were machined along horizontal and vertical directions from the as-deposited walls by wire electrode cutting. Sampling positions are shown schematically in Figure 2a. Before that, 10 mm of both ends of each wall were cut off and discarded and then machined as shown in Figure 2b. Tensile samples were machined in accordance with ISO 6892-1-2009 standard. Tensile tests were performed at room temperature on an electro-mechanical universal testing machine (Instron, Norwood, MA, USA) with a constant quasi-static velocity of 1 mm/min. The metallographic specimens were mounted in the thermal-set resin and grinded on abrasive papers from 400# to 2000# and then polished. The middle region of each sample containing several deposition layers was observed to measure the total number, mean pore diameter, and area percentage of pores using Imagine-Pro Plus software (IPP, 6.0, Media Cybernetics, MD, USA). Six pictures were taken continuously from the bottom to the top with a magnification of 50 times. Pores smaller than 5 μm are not counted because of the limitation of light microscopy. Afterwards, an anodizing film was formed in a 25 g/L fluoroboric acid solution using an ElectroMet 4 electrolytic polishing etchometer (Buehler, Lake County, IL, USA) at a voltage of 26 V for 1 min before microstructure observation. A light microscope with a Buehler OmniMet image analysis system was employed to reveal the microstructural morphology of the samples. A scanning electron microscope (SU3500, HITACHI, Tokyo, Japan and GeminiSEM-500, Carl Zeiss, Oberkochen, Germany) equipped with energy dispersive spectrometer (SEM-EDS, Oxford Instruments, Oxford, UK) was used to reveal the detailed microstructure and chemical compositions of the phases in the deposited 5183-Al wall. A multipurpose X-ray diffraction system (SmartLab 9 KW, Rigaku, Japan) with a Cu Kα source was used for phase analysis. 

## 3. Results and Discussion

### 3.1. Heat Input Evaluation of Different Arc Modes

**Figure 3:**
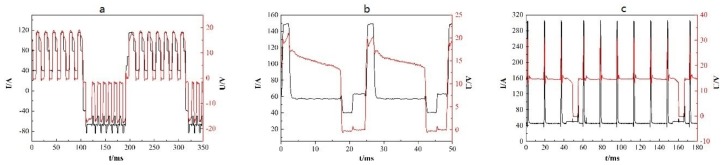
The arc current and voltage waveforms of (**a**) CMT+A, (**b**) CMT, and (**c**) CMT+P.

**Figure 4:**
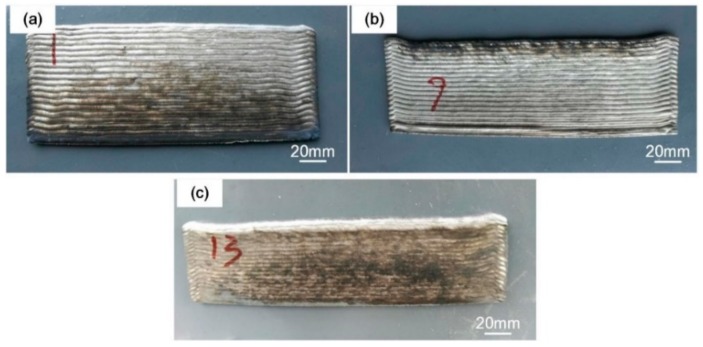
5183-Al thin wall-shaped samples deposited by (**a**) CMT+A, (**b**) CMT, and (**c**) CMT+P.

The heat input, the most important factor in producing desirable metallic parts for the WAAM process, should be appropriately controlled to ensure an excellent interlayer bonding and to decrease distortion due to heat cycling. In this research, three arc modes including conventional cold metal transfer (CMT), CMT+Pulse (CMT+P), and CMT+Advanced (CMT+A) were used to deposit the 5183-Al wire. The heat input of these three kinds of arc modes were calculated by the following equation [22,23]

(1) $$\mathrm{HI}=\frac{\int_{t_{1}}^{t_{2}}U_{i}I_{i}\mathrm{dt}}{t_{2}-t_{1}}/\mathrm{TS}$$

where HI is heat input, U is voltage, I is current, t is time, and TS is the travel speed. The voltage and current waveforms of the CMT, CMT+A, and CMT+P processes are shown in Figure 3. Calculated from the above equation, the heat inputs of CMT, CMT+A, and CMT+P process were 157.8 J/mm, 165.3 J/mm, and 185.4 J/mm, respectively. Obviously, the CMT+P process has the highest heat input during the WAAM process while the CMT mode has the lowest heat input. It can be seen from Figure 4 that all samples produced by the three modes had good surface appearance. The CMT+A manufactured sample shows the most smooth top surface, which indicates that 5183-Al can be effectively fabricated by the CMT+A process.

### 3.2. Porosity Characteristics

Porosity is commonly recognized as a major source of material discontinuity and defects in cast, wrought aluminum alloys and welded aluminum seams. This is the same for AM deposited aluminum alloys. The porosity formation mechanisms were mainly considered to be the precipitation of hydrogen gas or volumetric shrinkage upon solidification during AM [23]. The hydrogen sources were introduced by the contamination of the base metal or the filler wire. Hydrogen in the shielding gas is another possible source, even though it is now known that it contains very small amount of hydrogen during the welding process [24].

**Figure 5:**
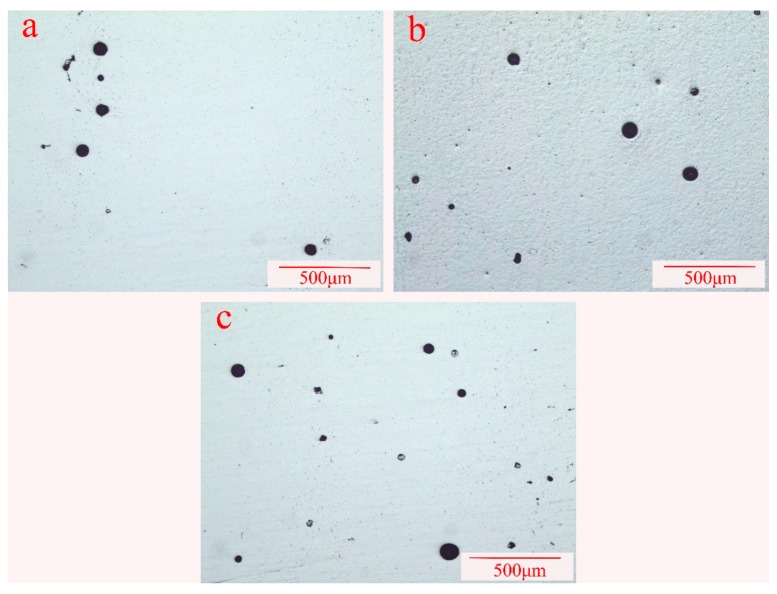
Light microscopy of pore distribution under three arc modes (**a**) CMT+A, (**b**) CMT, and (**c**) CMT+P.

**Figure 6:**
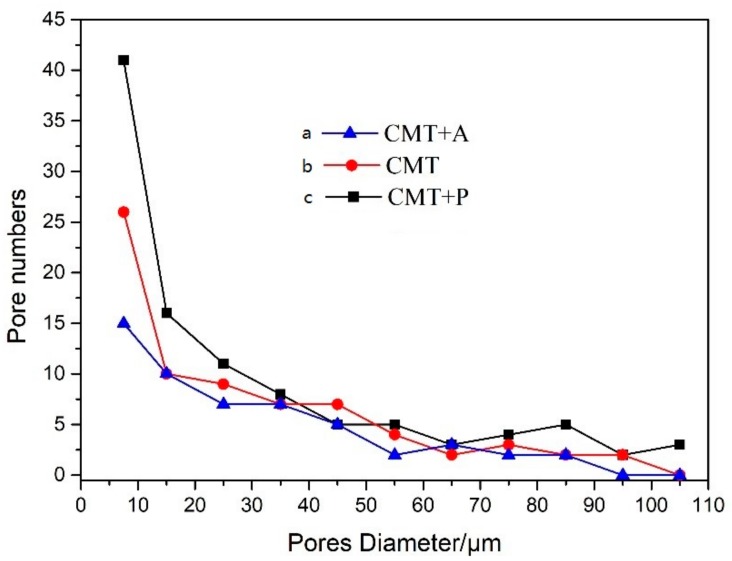
Pore numbers distribution classified by size with 10 μm interval (**a**) CMT+A, (**b**) CMT, and (**c**) CMT+P.

In this research, porosity was measured in the middle section of each wall-shaped sample under the light microscope without being etched. The micro-morphology of the porosity in the samples processed by the CMT+A, CMT, and CMT+P arc modes are shown in Figure 5. Figure 6 reveals the pore numbers’ distribution assorted by pore sizes with an interval of 10 μm. Pores larger than 100 μm were counted in the range of 100–110 μm in Figure 6, where shows that pore numbers decrease with increasing pore size. In particular, the numbers of pores that are greater than 100 μm in CMT+P sample is larger than that in the CMT and CMT+A samples. Table 2 is the analysis results of pores with different deposition modes. The mean diameter of pores in the CMT+P sample is 30.87 μm and the largest pore diameter achieves 129.16 μm. The average pores diameter and largest pore diameter of the CMT sample are 32.96 μm and 108.42 μm, respectively. The average pore diameter, 29.42 μm, of the CMT+A sample and its largest pore diameter 85.17 μm are smaller than the samples processed by the other two arc modes. The area percentages of pores in the CMT+A, CMT, and CMT+P samples are 0.36, 0.63, and 0.85, respectively. It can be concluded that the arc mode has a significant effect on pore formation. Pore formation is more sensitive during the CMT+P process. The largest pore size and the area percentage of pores in the sample processed by this process are significantly higher than samples built by the other two arc modes.

During the WAAM process, different welding arc modes means various heat inputs. As the heat input increases, both the pore size number become larger. Previous studies showed that the formation of pores is caused by the dramatic change of hydrogen solubility in the aluminum pool during the solidification process. Presence of hydrogen during the welding process is mainly produced by the filler wire, ambient atmosphere, and the base metal [25]. In this study, the deposition parameters, including Wire Feeding Speed (WFS), Travelling Speed (TS), and shielding gas flow, were kept identical for all three arc modes. Therefore, the pore formation can only be related to the arc modes, which leads to different deposition processes [26]. Previous research found that the variable polarity exerts a strong electromagnetic force to stir the pool and accelerate hydrogen bubbles escaping from the pool [13]. This is consistent with the statistical results that the pore number in the CMT+A sample is less than that in the CMT+P and CMT samples. In addition to that, the negative polarity plays a role of cathodic cleaning to remove aluminum oxides on the wire surface during the CMT+A process. For the CMT+P process, the droplets are refined by the pulses. This increases the specific surface area of droplets with capability to absorb hydrogen, enlarging the hydrogen pore sensitivity [22,27]. Previous studies also reported that solidification interface, secondary particles, dislocations, and grain boundaries can be used as heterogeneous nucleation sites for pores formation [28]. Therefore, porosity has a close relationship with the solidification process. Figure 6a shows the coarse columnar grain structure in the CMT+P sample. When the thin-wall sample is deposited layer by layer, the upper surface of the latter layer is always re-melted. The coarser re-melted solidification structure grows up across the layer boundaries. Moreover, the coarser column grain structure prevents floating up of hydrogen bubbles, which are trapped in the solidified structure [23,29]. Only small pores float up and combine into larger ones. Therefore, a great number of large pores are observed in the sample manufactured by the CMT+P process.

### 3.3. Microstructure Characteristics

**Figure 7:**
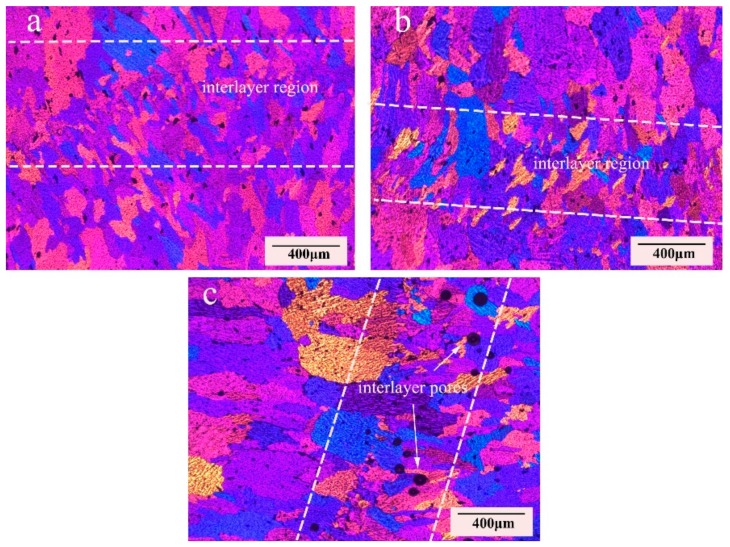
Metallographic observation of (**a**) CMT+A, (**b**) CMT, and (**c**) CMT+P arc modes.

Microstructures of 5183-Al alloys fabricated by different arc modes are shown in Figure 7. It can be seen clearly that the microstructure consists of interlayers of fine grain regions and internal layers of column grain zones both with CMT and CMT+A arc modes. There is a clear boundary between the deposition layers, as indicted by the dashed white lines. However, the interlayers’ fine grain can hardly be observed in the CMT+P sample. Column grains can be clearly observed in the CMT+P sample and the grain sizes are larger than that in the CMT and CMT+A samples. Besides, micropores can also be found at the interlayer regions.

Heat input has a significant influence on microstructural evolution during the deposition process. The continuous heat is transferred to the base metal/previous deposited layers along the wall with a high rate, forming a large temperature gradient through the wall. The growth of grains towards a direction with the fastest heat conduction velocity. Owing to the high temperature gradient, columnar grains are formed at the internal layers for all arc modes. In Figure 7a,b, smaller grains are formed at the internal layers for the CMT+A and CMT samples compared to the CMT+P mode. Because of the high energy input in the CMT+P process, the aluminum molten pool reaches a higher temperature with a lower solidification rate. Therefore, coarse grains grow up along the deposition direction, as shown in Figure 6c. In addition, the stirring effect of convert polarity exerts great electromagnetic force on the molten pool as mentioned above [20]. This force can break the dendrite tip in forming fractures, which act as nucleation sites of fine grains during solidification in the CMT+A arc mode. 

**Figure 8:**
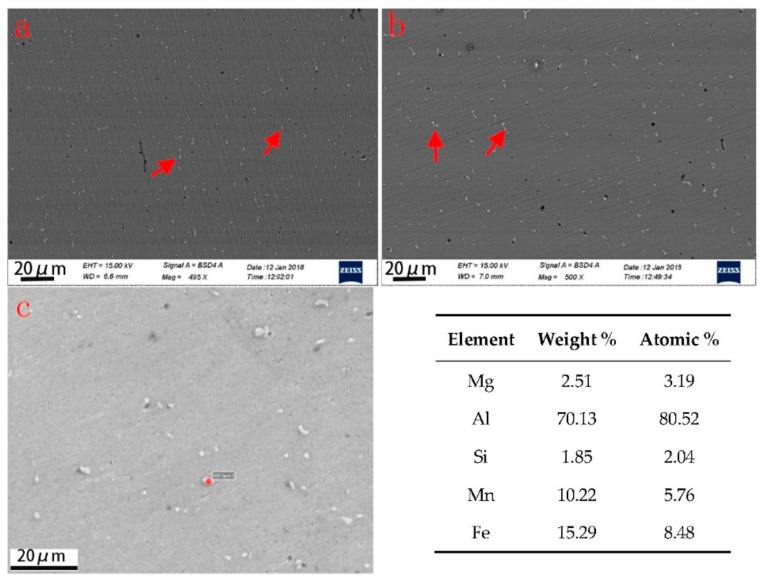
Secondary particles’ distribution in the (**a**) CMT+A and (**b**) CMT+P samples, (**c**) Chemical composition of secondary phase in the CMT+A sample.

**Figure 9:**
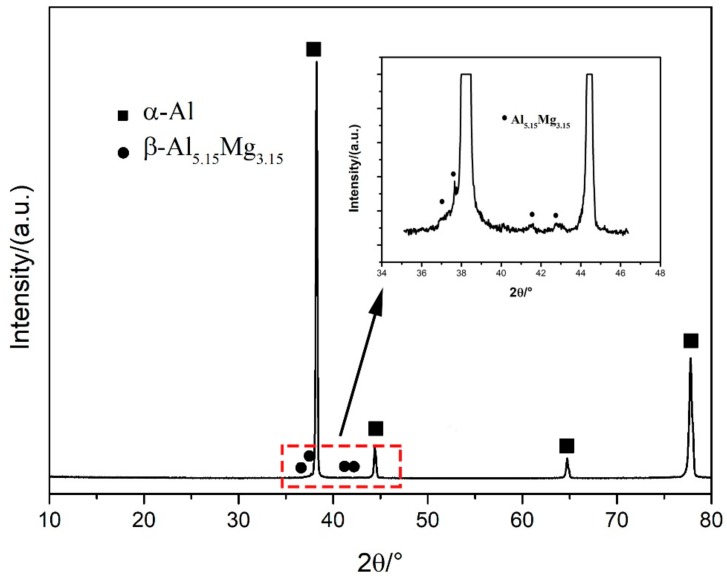
XRD pattern of the sample deposited by the CMT+A process.

In Figure 8, it can be seen that a large number of white particles are distributed uniformly in the matrix. As for Al-Mg alloys, the secondary phases could be Al_5_Mg_8_(Al_3_Mg_2_), Mg_2_Si, and (Mn, Fe)Al_6_ intermetallic compounds with various solidification processes [30]. Secondary phases precipitate from the melt of the welding seam with different morphologies and constitutions during the traditional welding process. For the three arc modes used in this study, the WAAM-deposited metallic walls are characterized by an Al matrix and secondary phases. Secondary particles distribute uniformly in the Al matrix with a dimension less than 10 μm for all the three arc modes. The chemical composition of the white particles in the CMT+A sample, which was analyzed and shown in Figure 8c, contains Al, Mg, Si, and Fe elements. This may be the Al_8_Mg_5_ phase and Mn-containing Al(FeMn)Si or Al_6_(MnFe) phases upon previous studies [31]. The XRD spectrum obtained from the CMT+A sample indicates that the β-Al_5.15_Mg_3.15_ phase is found in the alloy (see inset in Figure 9). These observations proved the white particles to be the β-Al_8_Mg_5_ phase. The particles in the CMT+P arc mode mainly exhibit a short rod-like morphology while fine particles are observed in the CMT+A process, as indicated by the arrows in Figure 8a. It is well established that the solute redistribution occurring during the solidification process is responsible for element microsegregation [32]. In the Al–Mg binary system, a eutectic reaction occurs at ~450 °C leading to the formation of α-Al and the β-phase Al_8_Mg_5_. WAAM is a process with high cooling and fast solidification rate. During solidification, Mg elements tend to segregate to the inter-dendritic region and grain boundaries [33,34]. Higher heat input of the CMT+P arc mode causes a lower solidification speed than that for the CMT+A and CMT processes [35]. Therefore, particles in the CMT+P sample are easier to be generated and grown up, leading to slightly less Mg content in the matrix and weaker solute strengthening effect. The size of secondary phases in the CMT+P sample is a little larger than that in the CMT+A sample.

### 3.4. Mechanical Properties of Different Welding Modes

**Figure 10:**
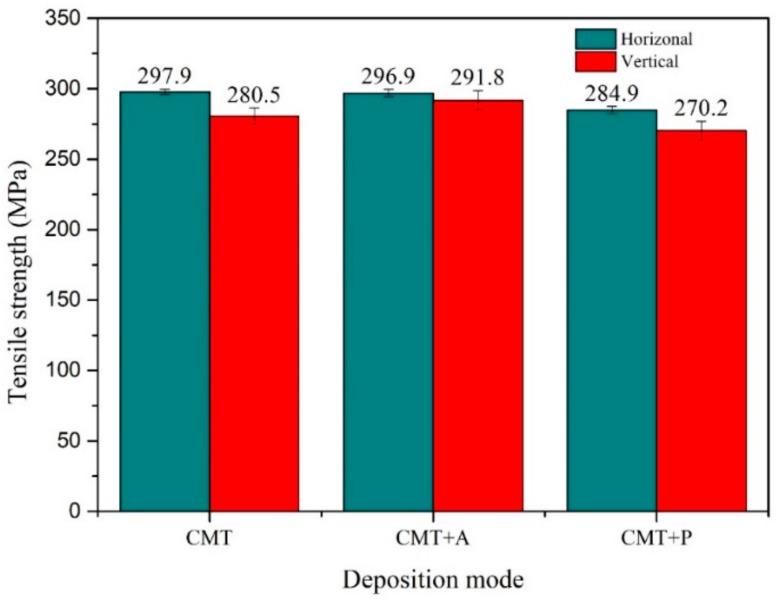
Horizontal (H) and vertical (V) tensile strengths for the three arc modes.

Tensile strengths along the horizontal and vertical directions of the samples built by different arc modes are presented in Figure 10. Each reported value is an averaged value of three test results. The horizontal and vertical tensile strengths for the CMT+A arc mode are 296.9 MPa and 291.8 MPa, respectively. It indicates that the tensile strength has little anisotropy under the CMT+A deposition process. The horizontal and vertical tensile strengths of the CMT+P processed samples are 284.9 MPa and 270.2 MPa, respectively. Larger difference between the two directions is found for the CMT samples, whose horizontal and vertical tensile strength are 297.9 MPa and 280.5 MPa, respectively.

Mechanical properties of aluminum alloys are sensitive to the microstructure and the presence of micropores in the alloy. As the 5183-Al alloy is a non-heat treatable material, solid solution strengthening, grain boundary strengthening, and precipitation strengthening have different effects on strength enhancement. Solid solution strengthening plays the most important role, followed by grain boundary strengthening and finally precipitation strengthening. Acquired by EDS, the average Mg content in the CMT+A and CMT+P samples are 5.9 wt.% and 5.67 wt.%. Therefore, the strengthening effect of Mg in CMT+A sample is the strongest and the tensile property is the best among all the samples. 

As mentioned above, the interlayer region is a weak position for the deposited parts. The CMT+A and CMT samples exhibit a fine grain structure at the interlayer zone. This is beneficial to improve the mechanical property along the vertical direction of the CMT+A sample. However, grain growth at the internal layer exhibits different characteristics for the CMT and CMT+P samples. When load is exerted on the tensile sample along the horizontal direction, the fine grain boundaries of the CMT+A sample acted as obstacles to hinder dislocation sliding [36]. This could decrease the anisotropy of the CMT+A sample. However, the CMT+P sample shows large column grains, i.e., smaller grain boundary density along the vertical direction, the blocking effects along this direction will be decreased. This may further weaken the tensile strength of this alloy. This may be one reason to explain the lowest tensile strength along the vertical direction for the CMT+P sample. However, it should be noted that the strengthening effect by refining grain size at this scale (>100 μm) is relatively low for Al alloys [37]. In addition, the reduced content of Mg solute atoms in the matrix due to precipitation according to the EDS results, as stated earlier, is perhaps another reason for the lower strength of the CMT+P samples. However, the major contribution to the difference of mechanical properties is related to the large amount of pores.

**Figure 11:**
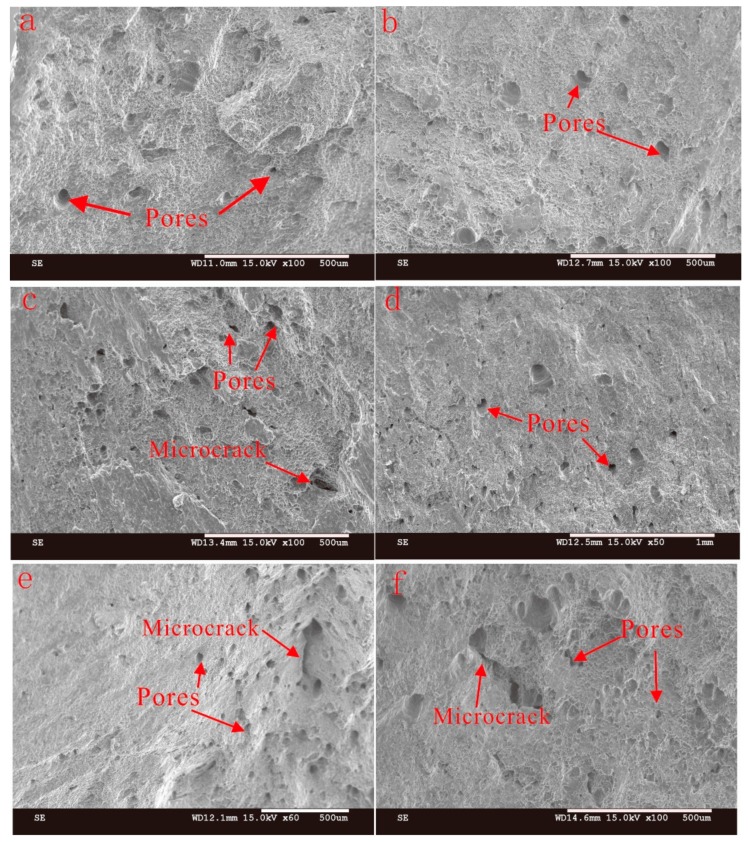
The fracture morphologies of CMT+A (**a**,**b**), CMT (**c**,**d**), and CMT+P (**e**,**f**) in the vertical (**a**,**c**,**e**) and horizontal (**b**,**d**,**f**) direction.

The presence of pores can be clearly seen from Figure 5 and Figure 11, which show the fracture morphologies for the samples fabricated with three arc modes. It can be found that test samples yield with a ductile failure appearance, showing predominant presence of dimples with tearing morphology in the deposition metal in Figure 11. As mentioned above, the white secondary particles distribute uniformly in the Al-matrix. Second phase particles are known to induce fracture [38]. However, there are almost no large particles on the fracture surface, this is probably due to their relatively smaller size (<10 μm) as compared to the pore sizes (>30 μm). Pores and microcracks are visible on the fracture surface, especially for the CMT+P sample. Microcracks may originate from the micropores and then join up together leading to the failure of metal parts [39]. Pores and microcracks can also be observed in CMT and CMT+P samples. The cracks may initiate from micropores and then expand to macrocracks. As discussed above, the pore area percentage and pore numbers of the CMT+P samples are obviously greater than that of the CMT+A and CMT samples. This indicates that pores are more sensitive to the applied stress [40] and can significantly reduce the tensile strength. More large pores are visible along the interlayer regions, especially in the CMT+P sample (see Figure 7c). This leads to lower strength in the vertical direction for the samples processed by the three arc modes. Based on the above discussion, it can be concluded that the CMT+A arc mode, which forms the smallest pore area percentage, is the most suitable arc mode for producing high quality 5183-Al metal parts.

**Figure 12:**
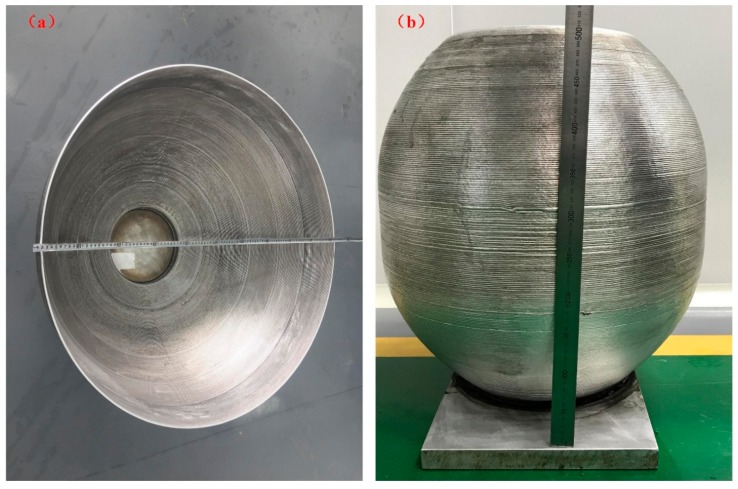
Large metal parts fabricated by CMT+A arc mode (**a**), with diameter of 1000 mm; (**b**), with diameter of 400 mm).

In addition to the superior strengthen of the CMT+A samples, Figure 12 shows several large 5183 aluminium parts (≈1 m) with a deposition rate of about 1 kg/h manufactured by the CMT+A arc mode. It can be seen from the figures that there is no obvious defect on the surface. Excellent dimensional uniformity is achieved, proving that the CMT+A arc mode is suitable for depositing 5183-Al.

## 4. Conclusions

In this research, the pore distribution, microstructure, and mechanical properties of 5183-Al thin-walled structures were systematically investigated. The following conclusions can be drawn based on the statistical analyses and experiments presented in this paper:

(1) The microstructure of CMT and CMT+A samples consists of interlayer fine grain region and layer column grains zone. The highest heat input of the CMT+P process contributes to production of column grains. Columnar grains of the CMT+P sample at internal layers are larger than that for the CMT+A and CMT samples.
(2) The pore area fraction in the CMT+P sample is the largest and the CMT+A sample is the smallest. Pore formation of the CMT+P process is mainly caused by the large heat input during deposition. The formed large column grain morphology prevents the escaping of pores from the molten pool.
(3) The best mechanical property greater than 290 MPa is obtained from the CMT+A arc mode compared with CMT and CMT+P arc modes. The tensile strength of the horizontal and vertical direction is more consistent for the CMT+A mode than the other two arc modes. The successfully manufactured large aluminium parts prove that the CMT+A arc mode is suitable for building 5183-Al components.

## Figures and Tables

**Table 1:** Chemical compositions of the filling wire and the base metal.

Alloys	Chemical Composition (wt.%)									
	Si	Fe	Cu	Mn	Mg	Cr	Zn	Ti	Be	Al
5183-Al	0.4	0.4	0.1	0.5–1	4.3–5.2	0.05–0.25	0.25	0.15	0.0003	Bal.
5083-H112	0.4	0.4	0.1	0.4–0.1	4.0–4.9	0.05–0.25	0.25	0.15	--	

**Table 2:** Analysis results of pores with different deposition modes.

Deposition Mode	Pore Numbers	Mean Diameter (μm)	Area Percentage (%)
CMT	65	32.96	0.63 ± 0.016
CMT+P	96	30.87	0.85 ± 0.015
CMT+A	54	29.42	0.36 ± 0.008
